# Supplementary material for: Nicotinamide Riboside and Dihydronicotinic Acid Riboside Synergistically Increase Intracellular NAD+ by Generating Dihydronicotinamide Riboside
Source: Nutrients. 2022 Jul 1;14(13):2752. doi: 10.3390/nu14132752 (PMC9269339; doi:10.3390/nu14132752)
Supplement: Supplementary file 1 [file nutrients-14-02752-s001.zip › nutrients-1794881-supplementary.pdf]

SUPPLEMENTARY MATERIALS

FIGURE S1

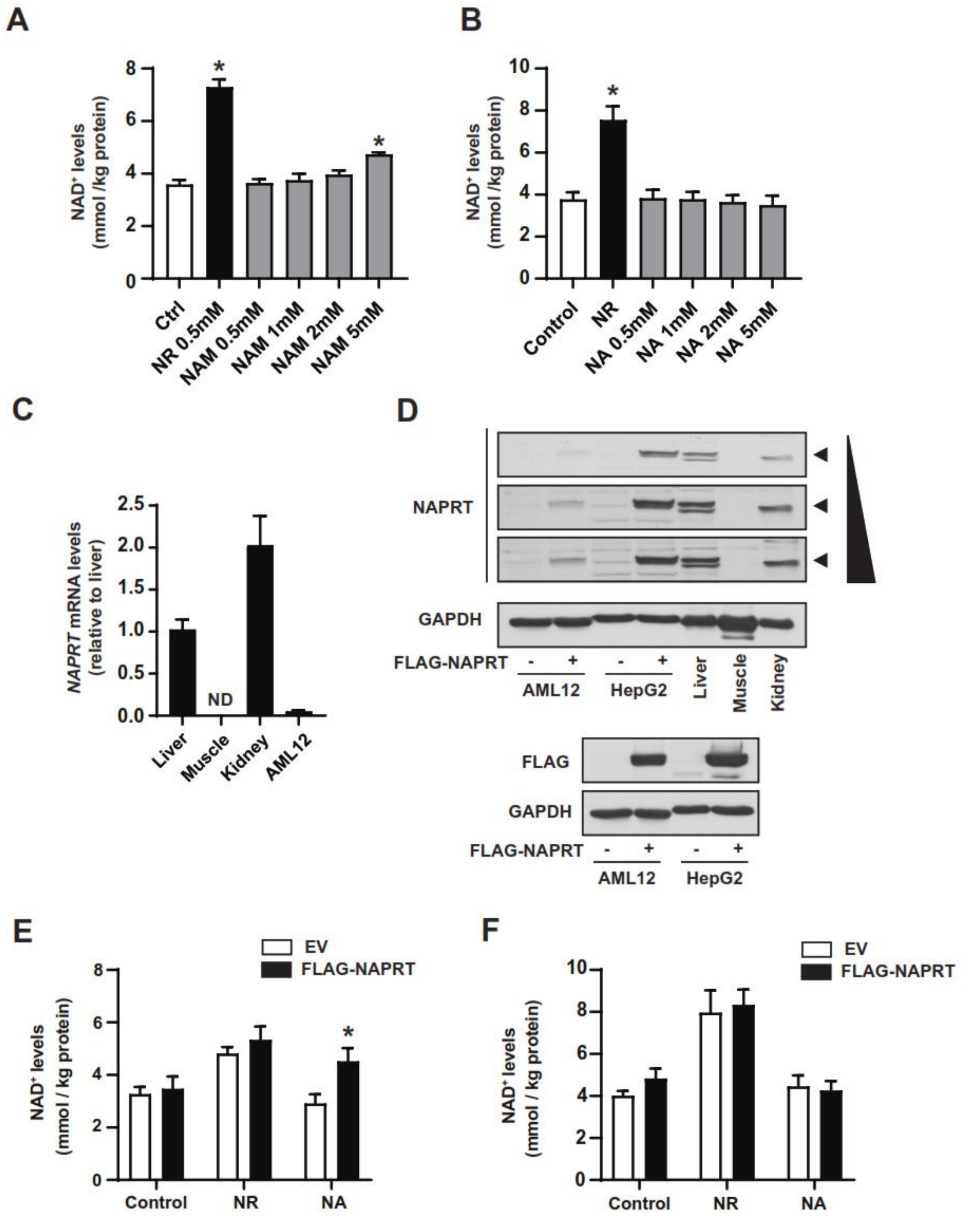

Figure S1. AML12 cells lack NAPRT activity. (A) AML12 were treated with PBS (as vehicle), NR (0.5 mM) or increasing doses of NAM for 2 hours and then acidic extracts were obtained to evaluate NAD^+ levels. (B) AML12 were treated with PBS (as vehicle), NR (0.5 mM) or

increasing doses of NA for 2 hours and then acidic extracts were obtained to evaluate NAD^+ levels. **(C)** NAPRT mRNA levels in AML12 and different mouse tissues, as measured by real time quantitative PCR. **(D)** AML12 and HepG2 cells were transfected with either empty vector or mouse NAPRT carrying a FLAG tag. Then, 48 hours later, cellular extracts were collected and western blot analyses were done, using mouse tissues as reference. **(E)** HepG2 cells were transfected with either empty vector or mouse NAPRT carrying a FLAG tag. Then, 48 hours later, cells were treated with 5 mM NA and, two hours later, NAD^+ levels were evaluated **(F)** as in (E), but using AML12 hepatocytes.

FIGURE S2

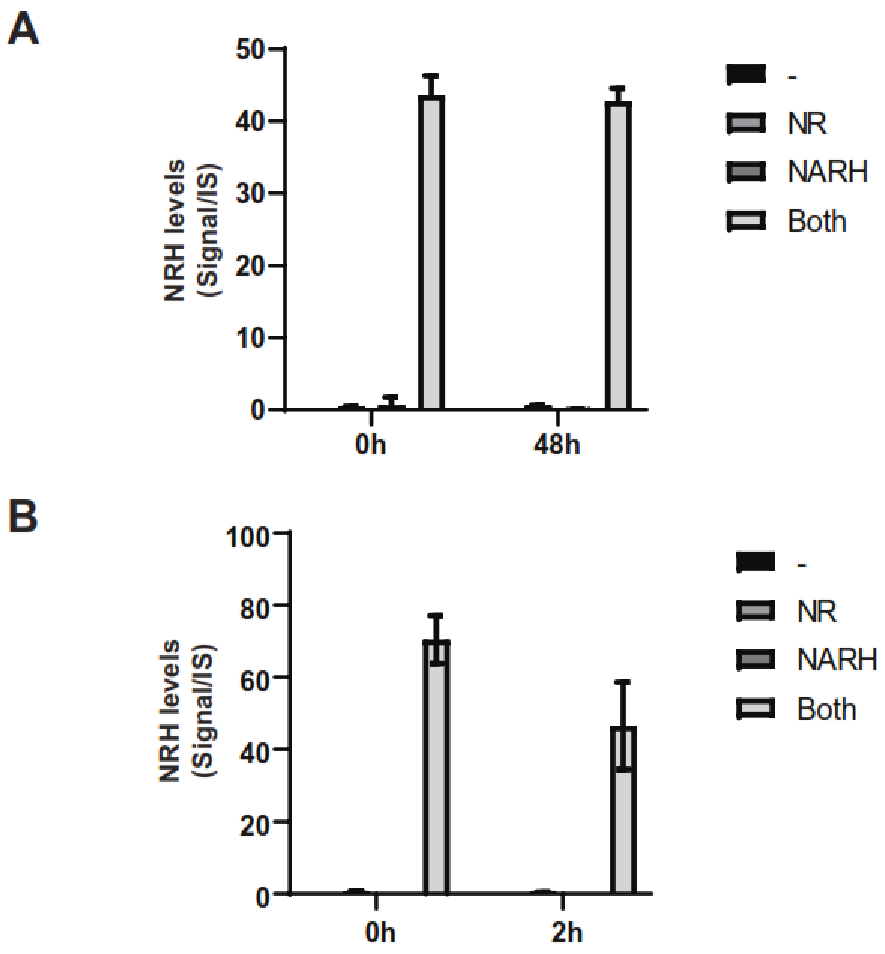

Figure S2. NR and NARH generate NRH extracellularly, in a non-enzymatic fashion. (A) NR (0.5 mM), NARH (0.5 mM) or both were spiked in water at room temperature and the levels of different metabolites were analyzed at t=0 and t=48 hours **(B)** NR (0.5 mM), NARH (0.5 mM) or both were spiked in mouse serum and the levels of different metabolites were analyzed at t=0 and t=2 hours. All values are presented as mean +/- SEM of n=3 experiments.

FIGURE S3

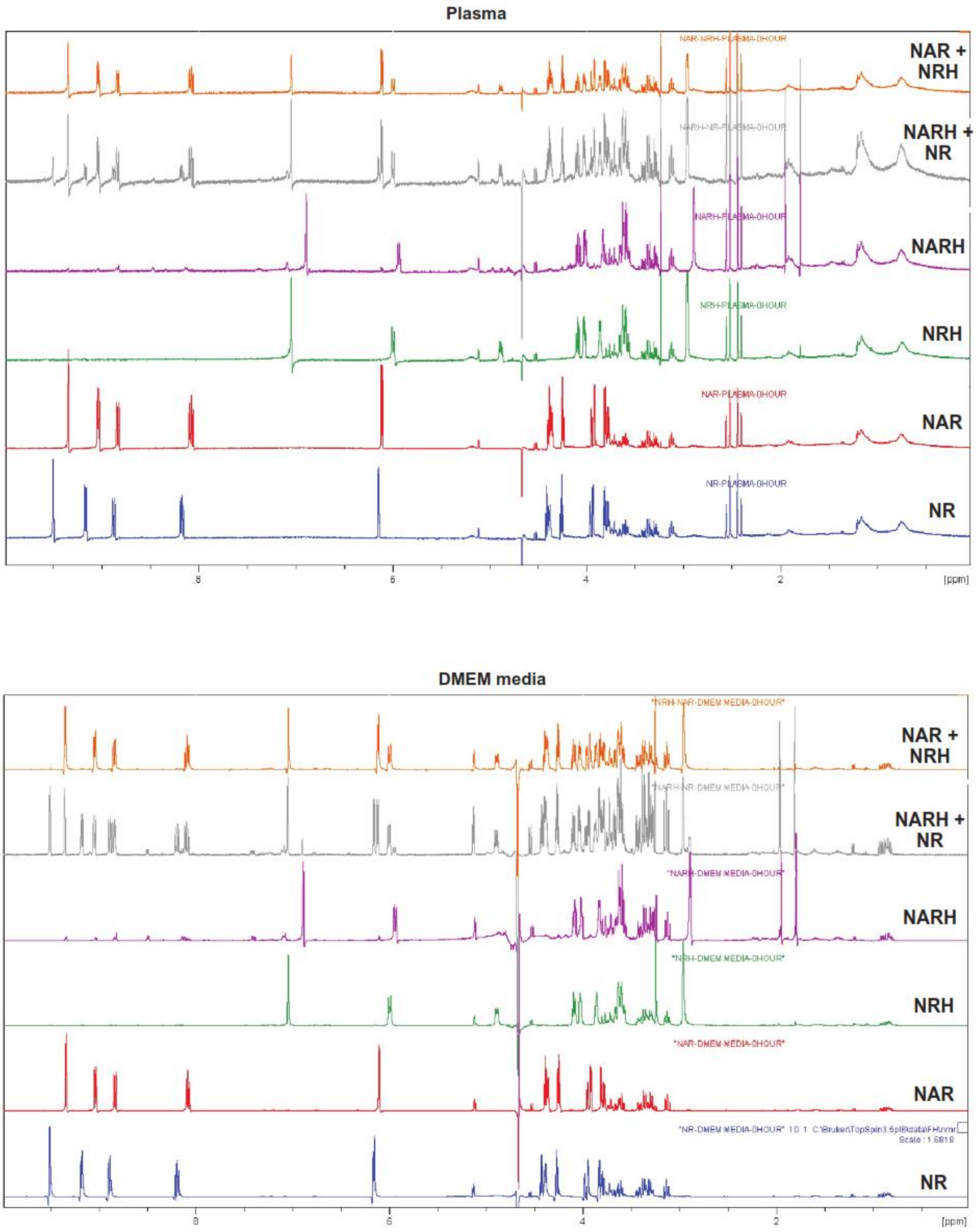

Figure S3. Transhydrogenation between NR and NARH in plasma and DMEM media. 1H NMR spectra of NR (NR chloride), NAR, NRH, NARH, NR+NARH and NRH+NAR (from top to bottom) in either human plasma (top panel) or DMEM media (bottom panel). In both cases, using 10% D_2O at mixing.

FIGURE S4

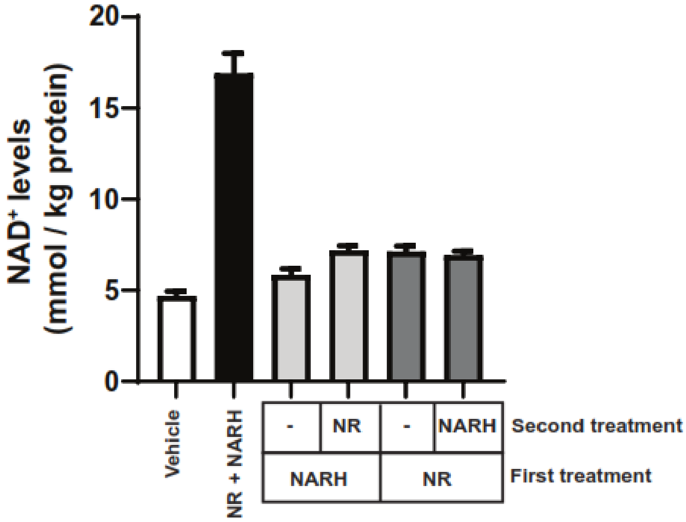

Figure S4. NR and NARH do not synergistically increase NAD^+ in cultured hepatocytes when used sequentially in a separate fashion. AML12 cells were incubated with either PBS (as vehicle), NR or NARH for 30 minutes. Then, cells were washed and incubated with similar or different treatments as indicated in the figure legend for additional 30 minutes. All values are presented as mean +/- SEM.

FiGURE S5

^{1}H NMR of NR in D_2O with chemical shifts allocation.

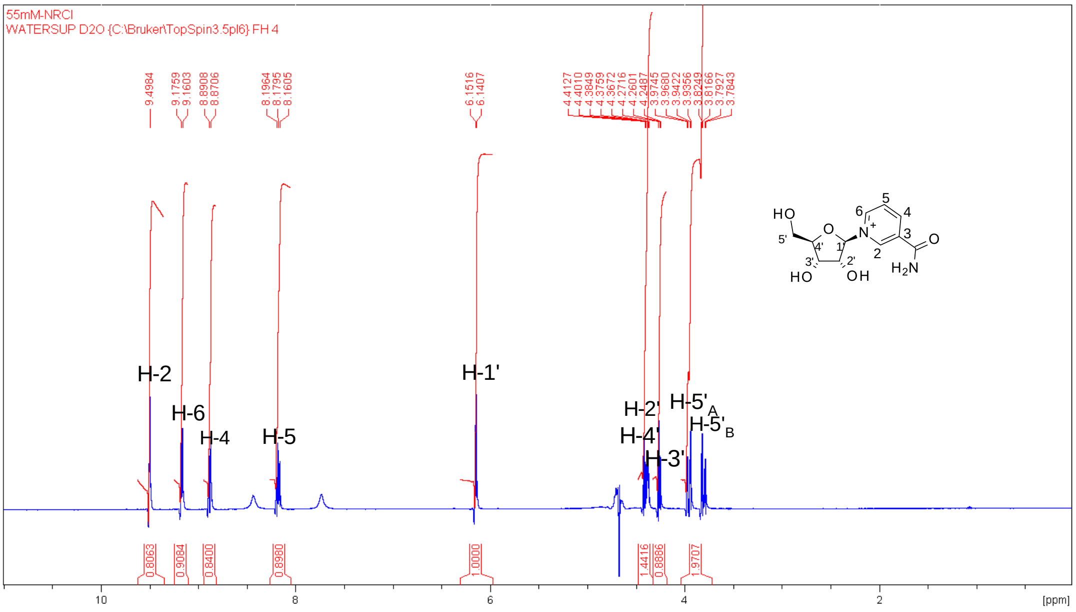

^{1}H NMR of NAR in D_2O

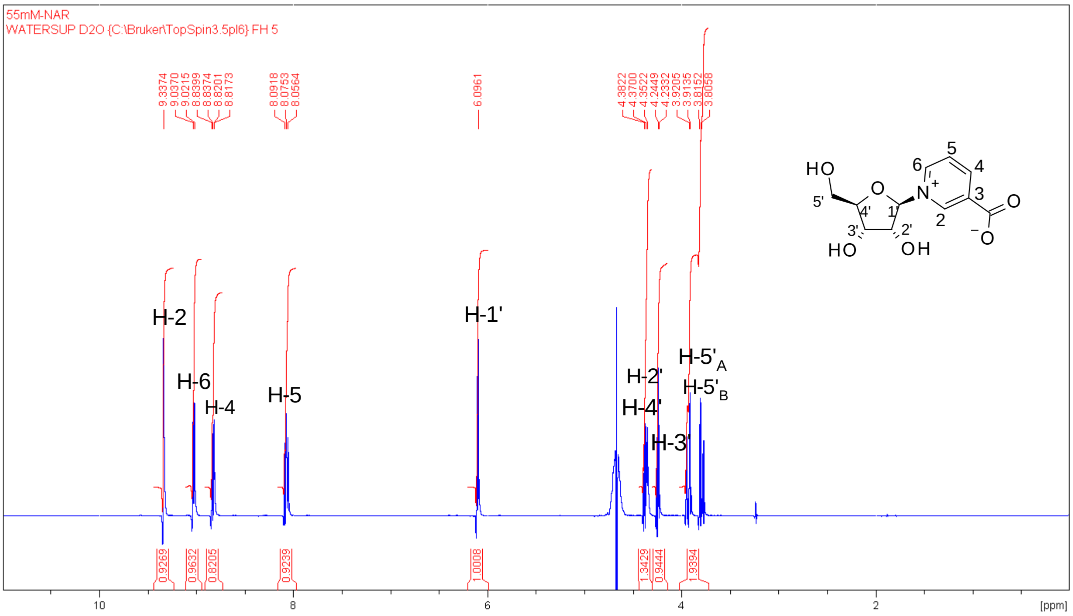

^{1}H NMR of NRH in D_2O

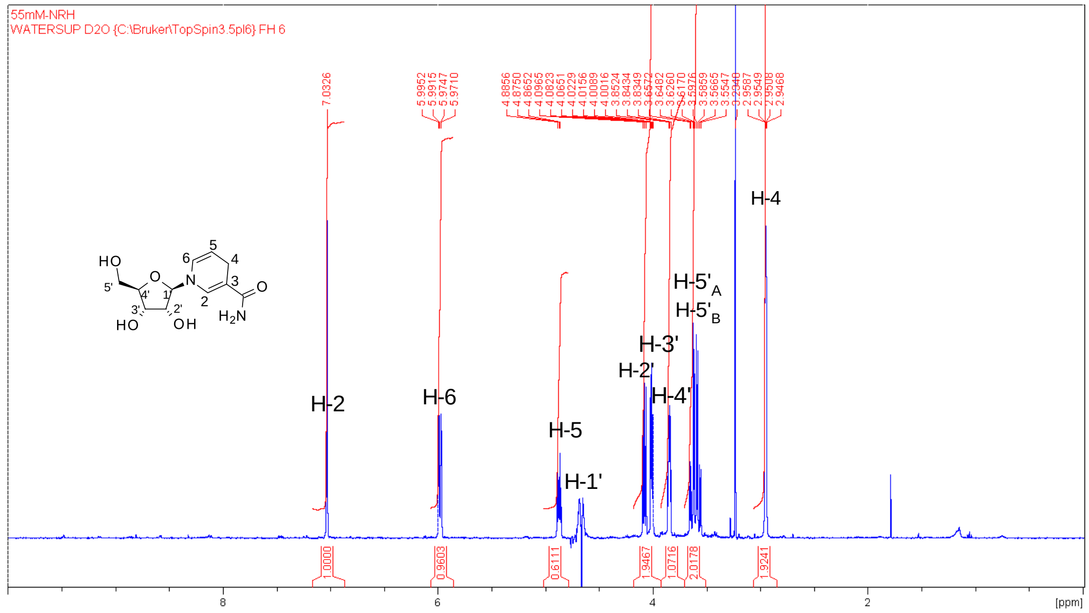

^{1}H NMR of NARH in D_2O

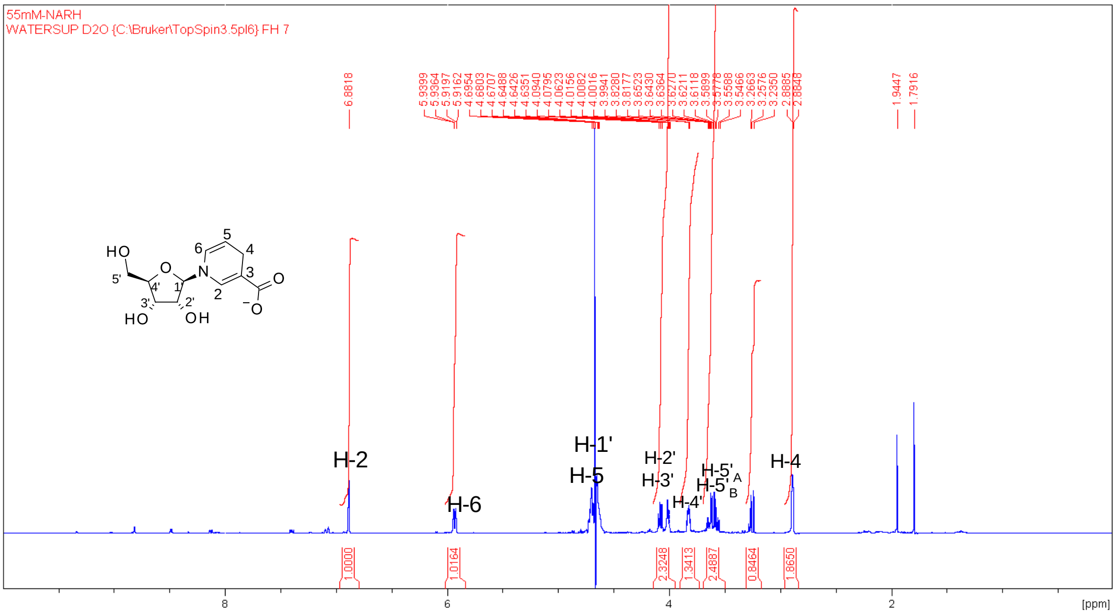

Figure S5. Chemical shift allocations for NAD^+ metabolites in 1H-NMR. From top to bottom panels, 1H NRM chemical shift allocations for nicotinamide riboside chloride (NR), nicotinic acid riboside (NAR), dihydronicotinamide riboside (NRH) and dihydronicotinic acid riboside (NARH)
